# Guidelines for the management of atrial fibrillation in adult patients admitted to an intensive care unit

**DOI:** 10.1016/j.aicoj.2026.100159

**Published:** 2026-09-14

**Authors:** Vincent Labbé, Nadia Aissaoui, Pierre Asfar, François Bagate, Simon Bourcier, Stephane Ederhy, Julie Helms, Sami Hraiech, Jérémie Joffre, Adrien Joseph, Khaldoun Kuteifan, Armand Mekontso Dessap, Sebastien Preau, Louise-Marie Jandeaux

**Affiliations:** a Department of Intensive Care, Hôpital Universitaire Bruxelles, Université Libre de Bruxelles, Bruxelles, Belgium; b Université Paris-Est, Groupe de Recherche Clinique GR05 CARMAS, Institut Mondor de Recherche Biomédicale, INSERM, Créteil, France; c Department of Cardiology, Georges Pompidou European Hospital, Assistance Publique des Hopitaux de Paris, Université Paris Cité, Paris, France; d Médecine Intensive Réanimation et Médecine Hyperbare, CHU Angers, Angers, France; e Service de Médecine Intensive Réanimation, Hôpitaux Universitaires Henri Mondor-Albert Chenevier, Département Médico-Universitaire Médecine, Assistance Publique des Hopitaux de Paris, Créteil, France; f Service de Médecine Intensive Réanimation, Groupe Hospitalier Sud Île-de-France, Melun, France; g Department of Pharmacology, Sorbonne Université, INSERM CIC-2503, AP-HP, Pitié-Salpêtrière Hospital, Paris, France; h Medicine Faculty, Medical Intensive Care Unit NHC, Strasbourg University, University Hospital Federation (FHU) TARGET, Strasbourg University Hospital, Strasbourg, France; i INSERM (French National Institute of Health and Medical Research, UMR 1260, Regenerative Nanomedicine (RNM), FMTS, Strasbourg, France; j Service de Médecine Intensive Réanimation, AP-HM, Hôpital Nord, Marseille, France; k Sorbonne Université, Assistance Publqiue Hôpitaux de Paris, Service de Médecine Intensive Réanimation, Hôpital Saint-Antoine, Paris, France; l INSERM UMR_S9380 Centre de Recherche Saint-Antoine, 75012 Paris, France; m Médecine Intensive Réanimation, Assistance Publique des Hôpitaux de Paris, Hôpital Ambroise Paré, Boulogne, France; n Service de Réanimation Médicale GHRMSA, 68100, Mulhouse, France; o Division of Intensive Care, Hôpital Roger Salengro, Centre Hospitalier Universitaire Lille, 59000, Lille, France

**Keywords:** Atrial fibrillation, Guidelines, Intensive care unit, Critically ill patients, Anticoagulation, Rhythm control, Rate control

## Abstract

Most existing recommendations for atrial fibrillation are derived from cardiology settings. However, atrial fibrillation is also highly prevalent among patients admitted to intensive care units and is associated with adverse clinical outcomes. Therefore, its management in this specific context warrants dedicated guidance. To address this need, The French Intensive Care Society has developed formalized expert recommendations using the Grading of Recommendations Assessment, Development, and Evaluation (GRADE) methodology.

Twenty-four questions addressing the management of atrial fibrillation were evaluated in adult patients admitted to intensive care units. Of these, twenty-one focused specifically on new-onset atrial fibrillation, one addressed pre-existing atrial fibrillation, and two were applicable to both conditions. The assessment encompassed the role of transthoracic and transesophageal echocardiography, the management of potentially reversible triggers (optimization of fluid status, magnesium supplementation, serum potassium targets, and screening for thyroid dysfunction…), strategies for mitigating hemodynamic risk (indications and modalities for rhythm and rate control), and the management of thrombotic risk (indications, regimens, and duration of therapeutic anticoagulation).

Eighteen recommendations were formulated, all of which achieved strong agreement among the expert panel. Only two recommendations were supported by a low level of evidence (GRADE 2+), whereas the remaining sixteen relied on evidence that could not be classified within the GRADE framework and were therefore issued as expert opinions. For six additional aspects of atrial fibrillation management, the experts concluded that the available evidence was insufficient to support the formulation of a reliable recommendation.

## Background

Atrial fibrillation (AF) is the most commonly observed arrhythmia among critically ill patients admitted to the intensive care unit (ICU), with a reported incidence ranging from 6% to 46% [[1], [2], [3], [4], [5], [6], [7]]. AF in ICU patients encompasses both new-onset atrial fibrillation (NOAF), defined as AF without a previous diagnosis of AF and considered clinically significant when lasting ≥30 seconds [8], and pre-existing AF, whether paroxysmal, persistent, or permanent [9,10]. These guidelines primarily focus on NOAF, as its pathophysiology and management in ICU patients may differ from those of AF encountered in conventional cardiology settings [9,10].

From a pathophysiological perspective, NOAF in ICU patients results from an interaction between patient-related predisposing factors, which constitute an underlying arrhythmogenic substrate (e.g., advanced age, hypertension, obesity, and cardiovascular or pulmonary comorbidities [1,2,4,11]), and triggers that may be directly reversible (e.g., electrolyte disturbances or hypovolemia) or related to the underlying acute illness (e.g., sepsis, thyroid dysfunction, acute coronary syndrome, or cardiac surgery) (Table 1). Accordingly, the onset of NOAF may reflect pre-existing remodeling, including fibrosis related to cardiovascular risk factors, that becomes clinically manifest when unmasked by the physiological stress associated with critical illness.

**Table 1:** Potentially reversible triggers associated with the occurrence of atrial fibrillation in the intensive care units (a non-exhaustive list).

Category	Potentially Reversible Triggers
Directly reversible factors	Electrolyte imbalances (magnesium, potassium, sodium, calcium, phosphate); Hypoxemia; Acidosis; Anemia; Vasopressors; Positive inotropes; Central line malposition; Fluid overload or deficit; Hyper-/hypothermia; Proarrhythmic drugs
Acute non-cardiac conditions	Non-cardiac surgery (perioperative, especially thoracic); Sepsis; Shock; Severe trauma; Pneumonia, pneumothorax, pulmonary embolism; Acute kidney injury; Acute neurological events (stroke, intracranial hypertension); Recreational drugs (methamphetamine, cocaine, opioids, cannabis); Drug overdose; Acute alcohol intoxication; Thyrotoxicosis
Acute cardiac conditions	Cardiac surgery or interventions (transcatheter aortic valve implantation, ablation); Acute coronary syndrome; Cardiomyopathies (ischemic and non-ischemic); Valvular heart disease; Myocarditis, pericarditis, endocarditis

NOAF in ICU patients may result in significant hemodynamic compromise and has been consistently associated with an increased risk of arterial thrombotic events (ATEs) and higher all-cause mortality [6,12,13]. Despite its clinical relevance, evidence guiding the management of the hemodynamic and thrombotic risks associated with NOAF in this setting remains limited, although the topic has recently garnered increased research interest. Consequently, the French Intensive Care Society (Société de Réanimation de Langue Française) initiated a formal guideline development process to provide specific guidance on the management of AF in ICU patients.

Based on a comprehensive review of the available evidence, these guidelines aim to define the role of echocardiography and provide guidance on the management of potentially reversible triggers, hemodynamic risk through rhythm- and rate-control strategies, and thrombotic risk through therapeutic anticoagulation.

## Methodology

In these guidelines, we included critically ill adult patients admitted to ICUs who exhibited AF at any point during their ICU stay. AF was categorized as new-onset in patients without a previous history of arrhythmia or as pre-existing in those with a documented history prior to the index hospitalization.

These recommendations are the result of the collaborative efforts of an expert panel convened by the French Intensive Care Society. The expert panel was composed of intensive care physicians working throughout France or abroad, with clinical experience in cardiac pathologies in the intensive care setting, recognized expertise in the field of AF, and participation in international clinical trials or publications in this research area. The expert panel was also selected to ensure balanced representation of university and non-university hospitals, Île-de-France and other French regions, and diverse opinion groups.

An independent steering committee affiliated with the French Intensive Care Society (the “Commission des Référentiels et de l’Evaluation”) was established. It was composed of three ICU physicians (SH, LMJ, KK) and one ICU nurse (MJ) with no intellectual or financial conflicts of interest, who were responsible for ensuring adherence to the methodology. The committee and the expert coordinator (VL) identified 24 key questions. Subsequently, experts were assigned to address each question. Following an initial expert group meeting, the questions were framed in accordance with the PICO format (Patient, Intervention, Comparison, Outcome; the PICO questions are listed in Supplemental File 1). A literature search was conducted using MEDLINE via PubMed and the Cochrane databases. Each expert conducted their own literature search using appropriate keywords. The results of these searches were available to the entire panel at the time of the recommendation were presented. Publications in English or French were included in the analysis, which focused on recent data in order of preference, from meta-analyses and RCTs to observational studies.

The experts used the modified Grading of Recommendations Assessment, Development and Evaluation (GRADE) methodology. Their task force defined key indexing terms, time limits, the target population, and the specific outcomes for which data were needed for the literature search. According to the GRADE methodology, a level of evidence was first assigned to each study identified by the search, based on study design and methodological quality. The experts then determined the overall level of evidence for each PICO question based on the level of evidence of the available studies, in accordance with the GRADE methodology, while also considering the consistency of results across the different studies and the risk/benefit ratio. A high overall level of evidence led to a “strong” recommendation (GRADE 1+, “should be done” or GRADE 1-, “should not be done”) and a moderate or a low level of evidence led to an “optional” recommendation (GRADE 2+, “should probably be done” or GRADE 2-, “should probably not be done”). In the absence of evidence, the recommendation was formulated as an expert opinion.

Proposed recommendations were presented and discussed one by one. The purpose of this process was not necessarily to reach a unique, convergent expert consensus on all of the proposals, but rather to define points of concordance, divergence, or indecision. Each recommendation was then evaluated by all experts, who individually rated their level of agreement on a scale ranging from 1 (complete disagreement) to 9 (complete agreement) using a “modified Delphi” method with anonymous voting. A score of 1–3 reflected disagreement with the recommendation, 4–6 indecision, and 7–9 agreement with the recommendation. A recommendation was considered approved if at least 50% of the experts agreed and no more than 20% disagreed. Strong agreement was defined as agreement by at least 70% of the experts. When strong agreement was not achieved, the recommendation was revised and subjected again to the rating process. Only expert opinions that obtained strong agreement were finally adopted.

## Results

The process resulted in the formulation of 18 recommendations, including 15 specific to ICU patients with NOAF, one specific to patients with pre-existing AF, and two applicable to both. For six PICOs, the experts concluded that the available evidence was insufficient to support the formulation of reliable recommendations. After two rounds of scoring, strong agreement was achieved for all 18 recommendations, sixteen of which were issued as expert opinions. Table 2 provides a summary of the recommendations.

**Table 2:** Summary of recommendations.




Abbreviations: AF, atrial fibrillation; NOAF, new-onset atrial fibrillation; R, recommendation; TTE, transthoracic echocardiography; TEE, transesophageal echocardiography.

## Section 1: The role of echocardiography

**Recommendation 1: In ICU patients with NOAF, the panel suggests performing systematic TTE to identify predisposing factors and potentially reversible triggers *(expert opinion, strong agreement)*.**

### Rationale

Outside the ICU setting, transthoracic echocardiography (TTE) is recommended as part of the initial evaluation of patients with NOAF. TTE provides clinically relevant information that may guide subsequent management decisions, particularly in patients presenting with significant changes in cardiovascular signs or symptoms [10].

In ICU patients with NOAF, TTE fulfills several key functions [14]. First, it may identify underlying cardiac conditions that predispose to AF, including left ventricular systolic or diastolic dysfunction, right ventricular dysfunction, cor pulmonale, right or left atrial (LA) dilatation, and valvular heart disease. Second, TTE may help identify potentially reversible triggers, such as hypovolemia, volume overload, pericardial effusion, or malposition of a central venous catheter [15]. Third, it enables assessment of the hemodynamic consequences of AF through evaluation of LV function, filling pressures, and stroke volume or its surrogate, the LV outflow tract velocity–time integral. Finally, TTE may inform hemodynamic management and anticoagulation decisions by identifying relevant structural abnormalities, including LV systolic dysfunction, LA dilatation, severe valvular heart disease such as mitral stenosis, intracardiac thrombi, and prosthetic valve dysfunction [14,16,17].

In ICU patients, no randomized controlled trials (RCTs) have evaluated the clinical benefit of systematic TTE in patients with NOAF. The available evidence is therefore primarily derived from prospective observational studies reporting the prevalence of clinically significant echocardiographic findings in this setting [4,5,[18], [19], [20], [21], [22], [23], [24]]. Reported abnormalities include valvular heart disease, pericardial effusion, hypovolemia, and LV systolic or diastolic dysfunction. The high prevalence of clinically relevant findings that may influence management supports the systematic use of TTE in ICU patients with NOAF.  

**Recommendation 2: The panel makes no recommendation regarding the systematic use of TEE in ICU patients with NOAF (no GRADE recommendation).**

### Rationale

Evidence specifically assessing the diagnostic and therapeutic impact of transesophageal echocardiography (TEE) in ICU patients with NOAF remains limited [23]. TEE may provide additional management-relevant information when detailed assessment of the left atrium (LA), and left atrial appendage (LAA), cardiac valves, or prosthetic devices is required. In the ICU setting, where patients are frequently intubated and sedated, TEE is generally feasible and provides high diagnostic accuracy for the detection of LA/LAA thrombi and other echocardiographic markers of thrombotic risk [23]. However, the incremental clinical benefit of routine TEE over TTE in ICU patients with NOAF has not been established. Accordingly, systematic TEE cannot be recommended, and a selective approach based on its anticipated impact on clinical management appears more appropriate. TEE may therefore be considered when exclusion of LA/LAA thrombus is required before cardioversion; when TTE provides inadequate imaging in patients with a high clinical suspicion of structural heart disease, potentially reversible triggers, or significant hemodynamic impact of AF; when infective endocarditis or prosthetic valve dysfunction is suspected; or when further characterization of an intracardiac thrombus or mass is expected to influence decisions regarding anticoagulation or cardioversion.

## Section 2: Trigger management

**Recommendation 3: The panel suggests not systematically administering fluid boluses to ICU patients with NOAF (expert opinion, strong agreement).**

### Rationale

Fluid bolus administration and fluid removal are reported in 19% (95% confidence interval [CI] 16–23) and 5% (95% CI 3–8) of ICU patients with NOAF, respectively [6]. Hypovolemia may act as a trigger for AF in ICU patients [15], and in such cases, rapid intravenous fluid bolus administration may contribute to ventricular rate control and facilitate restoration of sinus rhythm [25]. Conversely, an anteroposterior LA diameter >4 cm, as assessed by echocardiography, has been identified as one of the strongest predictors of incident AF in a cohort of ambulatory individuals, suggesting that volume overload may be a risk factor for NOAF [26]. Overall, there is limited pathophysiological and pharmacological evidence supporting the systematic administration of intravenous fluid boluses in patients with NOAF. Furthermore, to date, no RCTs have been published assessing whether a systematic administration of a rapid fluid bolus would improve outcomes in ICU patients with NOAF. Fluid management should therefore not follow a systematic approach but should instead be personalized based on repeated benefit-risk assessments [27]. In particular, fluid bolus administration should be reserved for patients in whom hypovolemia is considered a likely contributor to NOAF.  

**Recommendation 4: The panel makes no recommendation regarding the systematic administration of magnesium in ICU patients with NOAF (no GRADE recommendation).**

### Rationale

Magnesium infusion is used in 16% (95% CI 13–20) of ICU patients with NOAF [6]. Magnesium blocks slow inward calcium channels and potassium rectifier channels, thereby reducing sinus node automaticity, prolonging atrioventricular conduction and refractory periods, and exerting a class III antiarrhythmic effect [[28], [29], [30]]. Pathophysiological and pharmacological evidence supports its potential efficacy in controlling ventricular rate (rate control) and restoring sinus rhythm (rhythm control) in patients with AF. Low serum magnesium levels have been associated with the occurrence of AF in patients following cardiac surgery and in ambulatory populations [31,32]. Clinical data suggest that ICU patients with AF and hypomagnesemia may respond better to magnesium infusion than those with normal serum magnesium levels [33]. Since magnesium is primarily intracellular, serum levels may not accurately reflect total body stores [34], and intravenous magnesium may therefore be beneficial for rate or rhythm control, regardless of serum magnesium levels.

The expert panel analyzed all studies comparing magnesium as an adjunct to standard of care with a control strategy (i.e., standard of care ± placebo) in ICU patients. One single-center RCT compared magnesium sulfate plus amiodarone (n = 10) with amiodarone alone (n = 10) in ICU patients with NOAF following cardiac surgery [33]. No significant differences were observed at two hours in terms of rhythm control or treatment tolerance. Given the small sample size and the study’s specific focus on postoperative arrhythmias, these findings cannot be generalized to the broader ICU population.

A recent meta-analysis by Enayati et al. reviewed nine RCTs involving 1,048 non-postoperative patients with AF, comparing intravenous magnesium with placebo in addition to standard care for rate and rhythm control [35]. Magnesium sulfate (1.2 g–9 g) was infused over 5–30 min in patients with a normal mean serum magnesium level. Intravenous magnesium improved rate and rhythm control within 2–24 h after treatment. While flushing was more frequent, rates of hypotension were similar between groups. All studies excluded patients with arterial hypotension or renal dysfunction; participants were either cardiology outpatients or patients admitted to cardiology or emergency departments. ICU patients were not included in the analyzed studies. Consequently, the applicability of these findings to ICU patients is very limited, and the expert panel considered them outside the scope of these recommendations. A subgroup analysis of six RCTs in a previous meta-analysis by Ramesh et al. found that magnesium improved rhythm control in patients with AF and rapid ventricular response only at doses ≤ 5 g [36], suggesting that higher doses may not provide additional clinical benefit in terms of rhythm control.  

**Recommendation 5: In ICU patients with NOAF requiring cardioversion, the panel suggests that serum potassium levels should be maintained within the laboratory reference range (expert opinion, strong agreement).**

### Rationale

Outside ICUs, serum potassium measurement is part of the initial work-up for rhythm disorders, as hypokalemia is considered a potential trigger of AF [10,37]. Electrophysiological studies have shown that hypokalemia suppresses K^+^ channel conductance and inhibits Na^+^/K^+^-ATPase activity, thereby increasing intracellular Na^+^ and Ca^2+^ and activating Ca^2+^/calmodulin-dependent protein kinase (CaMK). CaMK enhances late Na^+^ and Ca²^+^ currents, reducing repolarization reserve and promoting early and delayed afterdepolarizations and automaticity [38,39].

In ICUs, hypokalemia and hyperkalemia have been reported in 11–25% [4,5,[40], [41], [42]] and 6–19% [4,5,42] of patients with NOAF, respectively. Additionally, small-sample studies have reported median or mean serum potassium concentrations at the time of NOAF ranging between 4.0 and 4.6 mmol/L [1,[43], [44], [45], [46]]. None of these studies found a significant association between dyskalemia and the risk of NOAF in ICU patients. In a prospective cohort of 67 ICU septic patients with NOAF, hypokalemia at AF onset was not associated with unsuccessful cardioversion [41]. Evidence regarding AF prevention for the present treatment question is indirect: outside ICUs, the perioperative TIGHT-K RCT subsequent to coronary artery bypass grafting found that potassium supplementation below 3.6 mmol/L was non-inferior to a 4.5 mmol/L threshold for AF prevention [47]. Consequently, available evidence does not support routinely targeting a serum potassium level ≥4.5 mmol/L either to prevent AF or to improve cardioversion success in ICU patients with NOAF.

Overall, clinical research is urgently needed to define the optimal serum potassium target for ICU patients with NOAF. These recommendations rely primarily on the pathophysiological understanding of the electrophysiological effects of dyskalemia.

**Recommendation 6: In ICU patients with NOAF, the panel suggests not systematically testing TSH, but rather testing it in the presence of one of the following: clinical features of hyper- or hypothyroidism, known thyroid hormone replacement or antithyroid therapy before ICU admission, planned amiodarone use, or cardioversion failure (expert opinion, strong agreement).**

### Rationale

Thyroid function assessment is recommended in cardiology guidelines as part of the initial work-up in all patients with AF [10,37]. Hyperthyroidism is a recognized trigger of AF. In animal studies, triiodothyronine (T3) has been shown to exert arrhythmogenic effects through mechanisms involving ion channels [48]. Pulmonary vein cardiomyocytes exposed to T3 have exhibited shorter action potential duration, leading to reduced refractoriness, facilitated formation of reentrant circuits, and an increased incidence of early and delayed afterdepolarizations [49]. Moreover, enhanced activity of beta-adrenoceptors in cardiac myocytes and atrial enlargement related to expanded blood volume may further contribute to AF in hyperthyroidism [50].

Outside ICUs, large-scale cohort studies have confirmed an association between hyperthyroidism and AF [[51], [52], [53]]. Conversely, hypothyroidism has not been clearly linked to AF in epidemiological studies, and pathophysiological data are conflicting [48,52,54,55]. In the Framingham Heart Study, a 10-year follow-up of 5,069 patients with hypothyroidism did not demonstrate an increased incidence of AF [54].

In ICU patients with NOAF, the relevance of routine thyroid function screening is debatable, especially insofar as common precipitants such as sepsis or systemic inflammation frequently coexist. A prior history of thyroid disease has been reported in up to 6% of ICU patients with NOAF [1,4,20], while the prevalence of newly identified thyroid dysfunction ranges from 0.5% to 14% when hormone levels are assessed at AF onset [5,6,12,42]. AF is almost invariably present in thyrotoxic heart disease, a rare but life-threatening complication of severe hyperthyroidism [56], and notably, 11% of ICU patients with severe hypothyroidism develop AF [57].

Given these observations, intensivists should measure TSH in ICU patients with NOAF when clinical features of thyroid dysfunction are present, particularly when no other reversible factors are apparent or when there is a prior history of thyroid disease. Awareness of thyroid status is likewise important when considering amiodarone, as hyperthyroidism may contraindicate its use [10]. However, when amiodarone is required urgently because of the patient’s hemodynamic condition, treatment need not be postponed until the TSH result is available.

## Section 3: Hemodynamic risk management (rate and rhythm control)

### Section 3A: Rhythm or rate control

**Recommendation 7: In ICU patients with NOAF during cardiogenic shock, the panel suggests favoring a rhythm-control strategy over a rate-control strategy, in addition to the management of potentially reversible triggers (expert opinion, strong agreement).**

#### Rationale

  In cardiogenic shock, the loss of atrioventricular synchrony and rapid ventricular response can be particularly detrimental to hemodynamics due to impaired baroreflex function [58] and diastolic as well as systolic dysfunction [59]. The restoration and maintenance of sinus rhythm in cases of severe tachycardia-induced cardiomyopathy with cardiogenic shock and atrial arrhythmias have been shown to result in significant improvements in both hemodynamic stability and echocardiographic parameters [60]. While no RCT has evaluated rate control versus rhythm control during cardiogenic shock, the restoration of atrioventricular synchrony appears physiologically appropriate in this setting.

**Recommendation 8A: In ICU patients with NOAF following cardiac surgery without hypotension or acute pulmonary edema, either rhythm control or rate control should probably be used, in addition to the management of potentially reversible triggers (GRADE 2+; strong agreement).**

**Recommendation 8B: Outside the postoperative cardiac surgery setting, in ICU patients with NOAF without hypotension or acute pulmonary edema, the panel makes no recommendation regarding whether a rhythm-control or rate-control strategy should be used in addition to the management of potentially reversible triggers (no GRADE recommendation).**

#### Rationale

  In an RCT including patients undergoing cardiac surgery, perioperative NOAF occurred in 695 patients (33% of those screened), among whom 523 hemodynamically stable patients were randomly assigned to either rate-control or rhythm-control groups [61]. The primary endpoint, the total number of hospitalization days within 60 days after randomization, was similar in the two groups (median, 5.1 days vs. 5.0 days; P = 0.76). There were no significant differences in the rate of death or serious adverse events, including thrombotic and bleeding events. Approximately 25% of patients in each group deviated from the assigned therapy, mainly due to drug ineffectiveness in the rate-control group or amiodarone side effects in the rhythm-control group. At 60 days, most patients in both groups had achieved sinus rhythm. This RCT suggests that rate-control and rhythm-control strategies yield similar outcomes in patients with NOAF without hypotension or acute pulmonary edema following cardiac surgery and that either strategy may be used in this context. The final choice of strategy should be guided by individual patient characteristics and tolerance to the medications used.

No RCT has compared these approaches in hemodynamically stable ICU patients outside the cardiac surgery setting. Therefore, no recommendation can be made for or against either rhythm control or rate control in this population.

**Recommendation 9: In ICU patients with NOAF during septic shock, the panel makes no recommendation regarding the use of a rhythm-control or rate-control strategies, in addition to the management of potentially reversible triggers (no GRADE recommendation).**

#### Rationale

  NOAF is the most common cardiac rhythm disturbance in ICU patients [1], with prevalence of 40% in septic shock , during which NOAF is associated with increased catecholamine requirements, in both dose and duration [5,7]. However, to date, no RCT has compared rate versus rhythm control alongside the management of potentially reversible triggers in septic shock patients with NOAF. Consequently, no recommendation can be made in this context. The lack of robust data from RCTs should encourage high- quality studies designed to assess the efficacy and safety of these hemodynamic strategies in this specific population.

### Section 3B: Modalities of rhythm control

**Recommendation 10A: In ICU patients with NOAF and concomitant cardiogenic shock or acute pulmonary edema requiring invasive mechanical ventilation, when urgent restoration of sinus rhythm is required, the panel suggests electrical rather than pharmacological cardioversion (expert opinion, strong agreement).**

**Recommendation 10B: In ICU patients with NOAF and concomitant acute pulmonary edema not requiring invasive mechanical ventilation, when urgent restoration of sinus rhythm is required, the panel suggests pharmacological rather than electrical cardioversion (expert opinion, strong agreement).**

#### Rationale

  A rhythm-control strategy aimed at restoring sinus rhythm can be achieved through synchronized external electrical shock (electrical cardioversion) or drugs (pharmacological cardioversion) [10,62]. To date, no RCTs have compared these two strategies in ICU patients with NOAF.

Theoretically, electrical cardioversion provides immediate restoration of sinus rhythm, whereas pharmacological cardioversion depends on the pharmacokinetics of the administered agent. In a recent systematic review involving 13,949 ICU patients with NOAF, pharmacological rhythm control was reported in 23 studies, including four RCTs, with success rates ranging from 18% to 94%. The time to sinus rhythm was inconsistently reported and ranged from 4 to 72 h [63]. Electrical cardioversion achieved immediate restoration of sinus rhythm in 48%–100% of patients [[64], [65], [66], [67]]; however, early relapse was common, with only 15%–31% of patients remaining in sinus rhythm 24 h after electrical cardioversion [40,[65], [66], [67]].

The choice between pharmacological and electrical cardioversion in ICU patients depends on the urgency of rhythm control and the risks entailed by each strategy.

Although cardiovascular complications, including stroke and rhythm disorders, are relatively rare [65,66,68], electrical cardioversion requires sedative agents, which may worsen hemodynamic or respiratory instability in ICU patients.

**Recommendation 11 A: In ICU patients with NOAF and sepsis without severe left ventricular systolic dysfunction, either amiodarone or propafenone should probably be used as the first-line pharmacological agent to restore sinus rhythm (GRADE 2+; strong agreement).**

**Recommendation 11B: In other ICU patients with NOAF, the panel suggests using amiodarone rather than other antiarrhythmic drugs as the first-line pharmacological agent to restore sinus rhythm (expert opinion, strong agreement).**

#### Rationale

  Data on the optimal first-line pharmacological agent for restoring sinus rhythm in ICU patients with NOAF remain limited.

An RCT including 209 septic shock patients with NOAF and LV ejection fraction greater than 35% reported that propafenone and amiodarone (both administered as an intravenous bolus followed by continuous infusion) yielded similar rates of rhythm control at 24 h (72.8% vs. 67.3%, P = 0.4) [69]. In a subgroup analysis, propafenone induced faster cardioversion (3.6 h vs. 8.5 h, P = 0.009) with fewer recurrences (50% vs. 76.8%, P = 0.001) in patients with a non-dilated LA (volume < 40 mL/m²) [17]. Conversely, in patients with a dilated LA, rhythm control occurred earlier with amiodarone (6.4 h vs. 18 h, P = 0.05) with comparable recurrence rates [69].

In a large multicenter cohort of ICU patients, intravenous amiodarone and beta-blockers showed similar efficacy for rhythm control at 24 h, and were superior to calcium channel blockers [70]. However, another propensity-matched cohort found that, compared with beta-blockers, amiodarone use was associated with higher mortality in septic patients [71]; these findings should nonetheless be interpreted cautiously due to potential confounding factors and limited representation of ICU patients.

A small single-center RCT involving 60 ICU patients with NOAF reported no significant difference in cardioversion within four hours between intravenous amiodarone (42.5%) and diltiazem (30%) [72]. Similarly, a prospective observational study found comparable cardioversion rates at 24 h (87.1% vs. 86.7%) [73].

Overall, amiodarone remains the most frequently used antiarrhythmic agent for NOAF cardioversion in ICUs, accounting for approximately two-thirds of cases [[3], [4], [5], [6], [7],^19^,^23^,^40^,^44^,^74^,^75^]. Its multimodal mechanism encompasses properties of all four Vaughan Williams classes, and it is not contraindicated in patients with structural heart disease or coronary artery disease [[76], [77], [78]].

**Recommendation 12A: In ICU patients with NOAF with cardiogenic shock or acute pulmonary edema for whom pharmacological cardioversion with amiodarone has been decided upon, the panel suggests using intravenous amiodarone with a loading dose followed by a continuous infusion (expert opinion, strong agreement).**

  **Recommendation 12B: In ICU patients with NOAF without cardiogenic shock or acute pulmonary edema for whom pharmacological cardioversion with amiodarone has been decided upon, the panel makes no recommendation regarding the route of administration (oral or intravenous) (**n**o GRADE recommendation).**

#### Rationale

  Amiodarone is characterized by a long and highly variable half-life, a large volume of distribution with affinity for adipose tissue, and extensive tissue uptake [79]. It can be administered either intravenously or via the enteral route, though, due to intestinal absorption and hepatic metabolism, its oral bioavailability is erratic and unpredictable (22%–86%) [80]. These pharmacokinetic properties explain the need for a prolonged and variable oral loading period to achieve effective antiarrhythmic concentrations. Conversely, the intravenous route offers a faster onset of action but may cause local and systemic adverse effects such as phlebitis, hypotension, and conduction disturbances [10]. According to ESC guidelines, the recommended regimen consists of a 300 mg loading dose infused over 30–60 minutes, followed by a continuous infusion of 900−1,200 mg over 24 h [10].

An open-label, non-randomized controlled study compared a single oral loading dose of 26 mg/kg) with an intravenous dose (a bolus of 5 mg/kg over 30 min followed by a continuous infusion of 10–15 mg/kg) of amiodarone over 24 h in 72 ICU patients with NOAF [81]. Cardioversion rates were similar (65% oral vs. 67% intravenous), and no major adverse events were reported.

A more recent RCT in 231 less critically ill patients with NOAF admitted to an acute cardiovascular unit likewise compared oral and intravenous amiodarone [82]. Cardioversion rates were comparable, but intravenous administration yielded faster conversion to sinus rhythm (12 h vs. 20 h) and was associated with a significantly greater decrease in arterial pressure compared than the oral route.

Further comparative studies are needed to determine the optimal route of administration in ICU patients. Uncertainty also persists regarding the duration required to maintain sinus rhythm and the clinical relevance of transitioning to oral or enteral therapy once hemodynamic stability has been achieved.

### Section 3C: Modalities of rate control

**Recommendation 13: When rate control is decided upon in patients with pre-existing AF or NOAF who have neither cardiogenic shock requiring a vasopressor and/or positive inotropic agent nor left and/or right ventricular systolic dysfunction, the panel suggests using beta-blockers rather than other classes of rate-control drugs, such as amiodarone, digoxin, or calcium channel blockers (Expert opinion, strong agreement).**

#### Rationale

In an RCT including 55 surgical ICU patients with NOAF without hypotension or LV systolic dysfunction, esmolol and diltiazem achieved comparable rate control at 2 and 12 hours. Cardioversion rates at 2 hours were higher with esmolol (59% vs. 33%), without any differences in hemodynamic tolerance or mortality [83].

Three large-scale retrospective studies have evaluated various rate-control regimens in ICU patients with AF. In a propensity score–matched cohort of 39,693 septic patients, initial treatment with beta-blockers was associated with significantly lower hospital mortality than treatment with calcium channel blockers (RR 0.92; 95% CI 0.86–0.97), including among patients receiving vasopressors. In the same cohort, amiodarone was associated with higher hospital mortality (42%) than beta-blockers (20.5%) (P < 0.001), and digoxin use was linked to higher mortality (25.7%) than beta-blockers use across all subgroups, including patients receiving vasopressor therapy and those with heart failure [71]. In a study of 666 ICU patients, beta-blockers achieved faster heart rate reduction within the first hour than amiodarone or digoxin. Rates of hypotension were similar across the beta-blocker, amiodarone, and calcium channel blocker groups, whereas digoxin was associated with fewer hypotensive episodes. No significant differences in in-hospital mortality were observed between beta-blockers and other agents [84]. Another retrospective study of 1,200 ICU patients with NOAF showed no significant differences between beta-blockers and amiodarone in rate control (adjusted HR 1.15 [0.91–1.46]) or hospital mortality (adjusted HR 1.03 [0.53–2.03]) [70].

When continuous infusion of an ultra-short-acting β-blocker with greater β1-selectivity is selected for rate control, no RCTs have directly compared esmolol with landiolol in ICU patients. However, a recent propensity score–matched study compared their efficacy in ICU patients, of whom approximately 10% had AF (9.6% in the landiolol group vs. 10.3% in the esmolol group), with the remainder presenting with sinus tachycardia. Landiolol yielded superior heart rate control without hemodynamic adverse events during the first 24 h and appeared to have a less negative impact on cardiac output than esmolol, as indicated by higher minimum ScvO₂ and lower maximal PCO₂ gap values [85].

**Recommendation 14: When rate control has been decided upon in patients with pre-existing AF or NOAF who have cardiogenic shock requiring a vasopressor and/or positive inotropic agent or left and/or right ventricular systolic dysfunction, the panel suggests using amiodarone rather than other classes of rate-control drugs, such as beta-blockers, digoxin, or calcium channel blockers (expert opinion, strong agreement).**

#### Rationale

  A single RCT compared intravenous amiodarone with diltiazem in cardiac ICU patients with NOAF, among whom 75% were mechanically ventilated and 75% were on vasopressors and/or inotropic support when they developed NOAF. Both agents achieved a similar reduction in heart rate within 4 hours. Cardioversion rates and mortality did not differ between groups; however, hypotension occurred more frequently with diltiazem [72].

No studies have directly compared amiodarone with other classes of rate-control drugs in patients with cardiogenic shock or LV systolic dysfunction. Based on expert consensus, amiodarone is suggested over other rate-control drugs because of its favorable safety profile in patients with impaired LV ejection fraction or cardiogenic shock [[76], [77], [78]]. Additionally, given the potential unreliability of enteral absorption in ICU patients, the intravenous route is suggested to ensure a rapid onset of action.

## Section 4: Thrombotic risk management (therapeutic anticoagulation)

**Recommendation 15A: In ICU patients with NOAF lasting less than 48 h who are not undergoing cardioversion, the panel suggests not initiating therapeutic anticoagulation (expert opinion, strong agreement).**

**Recommendation 15B: In ICU patients with NOAF lasting at least 48 h who are not undergoing cardioversion, the panel suggests not routinely using therapeutic anticoagulation (expert opinion, strong agreement)**

**Remark: In this case, the panel suggests deciding whether to initiate therapeutic anticoagulation based on the risk-benefit balance, taking into account the patient’s pro-thrombotic state and bleeding risk.**

### Rationale

In-hospital ATEs occur in approximately 3%–11% of ICU patients with NOAF [2,3,23,86]. In a large-scale retrospective cohort of nearly 50,000 patients with sepsis, the risk of in-hospital ischemic stroke was more than doubled among those with NOAF (OR, 2.70 [95% CI, 2.05–3.57]) [2]. In the FAST multicenter prospective study of 94 intubated septic patients with NOAF, 7% experienced at least one ATE within a median of six days from AF onset, including five ischemic strokes, one non-cerebral thromboembolism, and two LAA thrombi [23].

ATEs generally arise from systemic embolism secondary to LA/LAA thrombus formation, as explained by Virchow’s triad (stasis, endothelial dysfunction, and hypercoagulability) [87]. Other mechanisms may involve intrinsic cerebrovascular disease, alternative cardiac embolic sources, or aortic atherosclerosis [23]. However, the risk of LA/LAA thrombus formation appears to be minimal in ICU patients when NOAF lasts less than 48 h. In a prospective multicenter cohort of intubated septic patients, systematic TEE within 48 h revealed no LA/LAA thrombi [23], a finding consistent with cardiology data [11]. In a prospective cohort of 423 ICU patients, the incidence of in-hospital stroke was 7.6% in those with AF > 48 h and 3.8% in those with AF of shorter duration [86].

Major bleeding events appear to be more frequent. A 12.6% incidence of bleeding was reported in a large retrospective cohort of septic patients with NOAF [71]. In the FAST cohort, 19% of patients developed at least one major bleeding event after a median of 8.5 days, including life-threatening events in 11% [23].

In the absence of RCTs, genuine clinical uncertainty remains regarding the benefit of therapeutic anticoagulation in ICU patients with NOAF, whether lasting less than or more than 48 h, when pharmacological or electrical cardioversion is not indicated. Observational data suggest that more than one-third of such patients receive therapeutic anticoagulation [3,23,86,88]. However, in a large propensity score–matched cohort of septic patients with NOAF, therapeutic anticoagulation increased the risk of bleeding (RR, 1.21; 95% CI, 1.10–1.32) without reducing thromboembolic events (RR, 0.94; 95% CI, 0.77–1.15) [88]. Similarly, in a post hoc analysis of 308 ICU patients, therapeutic anticoagulation was administered to 32% within 48 h and to 10% after 48 h following NOAF onset. Early initiation of anticoagulation did not reduce the composite outcome of stroke and death (adjusted HR, 0.77, 95% CI 0.47–1.23), suggesting that it does not improve clinical outcomes in ICU patients with NOAF [89].

In the cardiological context, the 2024 ESC guidelines recommend immediate therapeutic anticoagulation for all patients with a CHA_2_DS_2_-VA score ≥2, which reflects high thromboembolic risk. Assessment of modifiable bleeding risk factors is advised as a means of ensuring safety, and a HAS-BLED score ≥3 should identify patients at high bleeding risk [10]. However, these scores have not been prospectively validated in ICU populations. In the FAST cohort, CHA_2_DS_2_-VASc and HAS-BLED scores were similar in patients with or without ATE or major bleeding events. Among the components of the CHA_2_DS_2_-VASc score, only a history of stroke was associated with the occurrence of ATEs in ICU patients [3].

Finally, the 2024 ESC guidelines emphasize that in the acute phase of sepsis (the leading trigger of NOAF in ICU patients), the risk–benefit balance of therapeutic anticoagulation remains uncertain [10]. Recent systematic reviews have likewise concluded that current evidence is insufficient to support routine anticoagulation for NOAF in ICU patients [62,90]. Well-designed RCTs are needed to clarify the efficacy and safety of anticoagulant therapy in this high-risk population.

  **Recommendation 16: In ICU patients with NOAF undergoing cardioversion, the panel makes no recommendation regarding the initiation of therapeutic anticoagulation during the peri-cardioversion period, regardless of the duration of NOAF (**< 48 h **or** ≥ 48 h**) (no GRADE recommendation).**

### Rationale

Data on the risk of peri-cardioversion ATE among ICU patients with NOAF remain scarce. In the FAST cohort of intubated septic patients with NOAF, 6% experienced an ATE within 28 days after successful pharmacological or electrical cardioversion. Only 22% of these patients were receiving therapeutic anticoagulation at the time of cardioversion [23,41].

In non-ICU patients, two main mechanisms have been proposed to explain the increased thrombotic risk associated with cardioversion: embolization of a pre-existing thrombus and LAA stunning [91,92]. LAA stunning results from a temporary loss of organized mechanical contraction [92], even after sinus rhythm has been restored, and is characterized by reduced LAA filling and emptying velocities on TEE [[93], [94], [95], [96]]. Based on these observations, the 2024 ESC guidelines recommend that patients undergoing cardioversion receive therapeutic anticoagulation as early as possible in the peri-cardioversion period [10,97]. In patients without thrombotic risk factors (CHA₂DS₂-VA = 0) who spontaneously convert to sinus rhythm within 24 h, therapeutic anticoagulation may be optional [10].

However, the same guidelines explicitly acknowledge the lack of prospective studies and RCTs specifically evaluating therapeutic anticoagulation strategies in ICU patients [10]. Applying a peri-cardioversion therapeutic anticoagulation strategy in the ICU settings raises concerns because of diffences in thrombotic and bleeding risk profiles. In the ICU setting, the bleeding risk associated with therapeutic anticoagulation appears substantially higher than that observed in cardiology wards [23,71,98]. In the FAST cohort, systematic TEE performed within 48 h of NOAF onset demonstrated no left atrial thrombus [23], and LAA dysfunction after cardioversion was rare (2%) [99]. Based on these observations, several intensivists have advocated for a conservative anticoagulation approach, with delayed initiation and shorter duration, aimed at balancing ATE prevention against bleeding risk, as compared with the approach outlined in the 2024 ESC guidelines [100].

In conclusion, available evidence is insufficient to support routine therapeutic anticoagulation at the time of cardioversion in ICU patients with NOAF. The lack of robust data highlights the need for well-designed studies to determine the optimal anticoagulation strategy in this population.

**Recommendation 17: In ICU patients with NOAF for whom therapeutic anticoagulation has been decided upon, the panel suggests using UFH rather than LMWH (expert opinion, strong agreement)**

### Rationale

The optimal choice between UFH (unfractionated heparin) and low-molecular-weight heparin (LMWH) in ICU patients with NOAF remains unclear because of a lack of evidence favoring one over the other. Several considerations may guide anticoagulation modality selection. Therapeutic anticoagulation inherently increases the risk of bleeding, especially in ICU patients. In this setting, UFH may be preferred due to its shorter half-life and the availability of a specific reversal agent (protamine sulfate) in case of bleeding complications. ICU patients frequently develop multiorgan failure, including acute kidney injury, which can affect drug clearance. In these scenarios, UFH is more manageable than LMWH because it can be readily monitored and does not rely on renal elimination. Heparin-induced thrombocytopenia is a rare but serious complication of heparin use. While the risk of HIT is higher with UFH compared with LMWH, the overall incidence of HIT in ICU patients is relatively low, ranging from 0.6%–2.6% for UFH versus 0.2%–0.3% for LMWH [101]. This makes the risk less of a limiting factor, especially when balanced against other considerations, including reversibility. Regular platelet count monitoring is essential for early detection and management of HIT.

Given the lack of evidence favoring either UFH or LMWH, no firm conclusion can be drawn regarding the superiority of one strategy over the other in terms of efficacy and safety outcomes. Furthermore, maintaining therapeutic anticoagulation with UFH may be challenging in critically ill patients, resulting in prolonged periods outside the therapeutic range. The panel nevertheless favors UFH because of practical considerations, including its short half-life, reversibility with protamine, and ease of management in the settings of acute kidney injury and a rapidly changing bleeding risk.

**Recommendation 18: In ICU patients with NOAF who have returned to sinus rhythm and in whom therapeutic anticoagulation has been initiated, the panel suggests early discontinuation of therapeutic anticoagulation after cardioversion unless a specific indication for continued therapeutic anticoagulation exists (e.g., intracardiac thrombus) (expert opinion, strong agreement).**

### Rationale

From a pathophysiological perspective, ICU patients have multiple risk factors for thrombosis related to age, comorbidities, and the underlying condition prompting ICU admission. Additionally, transient prothrombotic risk factors, such as sepsis, may further increase thrombotic risk. NOAF therefore occurs in a highly thrombogenic context, warranting consideration of therapeutic anticoagulation to reduce thrombotic risk [2,3,23,86].

Post-cardioversion LAA stunning is considered a major contributor to thrombus formation in the first few days following cardioversion in non-ICU patients [102]. Recovery of LAA contractility is gradual, often taking several days and up to four weeks [102,103]. These findings are consistent with clinical data showing that approximately 80% of ATEs occur within the first three days following cardioversion, and nearly all within the first 10 days [104]. Based on these observations in non-ICU patients, current ESC guidelines recommend peri-cardioversion therapeutic anticoagulation for at least four weeks [10,97]. In the ICU setting, however, LAA dysfunction appears uncommon. A sub-analysis of intubated ICU patients with sepsis reported a prevalence of only 2% of LAA dysfunction after restoration of sinus rhythm [99]. These findings suggest that LAA behavior in ICU patients may differ from that in chronic AF, potentially due to distinct patterns of atrial remodeling and endothelial dysfunction [105]. Moreover, ICU patients have an increased bleeding risk [23,88,106], which is further elevated by the administration of therapeutic anticoagulation [98].

Considering these factors, several intensivists have advocated for a conservative anticoagulation strategy involving a shorter duration of therapeutic anticoagulation when administered, with the goal of balancing ATE prevention against reduced risk of major bleeding events [100].

To date, no study has formally evaluated the benefit-risk ratio of anticoagulation therapy for NOAF in ICU patients, and the optimal duration of anticoagulation following restoration of sinus rhythm remains unknown.

**Recommendation 19: Except in patients at very high thrombotic risk (e.g., multiple previous strokes, a mechanical heart valve, mitral stenosis, or an intracardiac thrombus), the panel suggests not continuing therapeutic anticoagulation in ICU patients with pre-existing AF during the acute phase of critical illness (expert opinion, strong agreement).**

### Rationale

Decision-making regarding therapeutic anticoagulation for pre-existing AF in ICUs is particularly challenging [107] due to alterations in the coagulation cascade, organ failure, sepsis [108], invasive support therapies, and surgery, all of which may increase both thrombotic [2,109] and bleeding risks [23,88,106,110]. Current cardiology guidelines on anticoagulation in AF may not be directly applicable to the critical care setting [10]. The decision to continue or temporarily discontinue therapeutic anticoagulation should therefore be individualized, considering patient characteristics, comorbidities, bleeding risk, ongoing organ dysfunction, invasive procedures, and the complexity of the acute illness.

The largest available observational study was a nationwide retrospective study of patients hospitalized with sepsis and concomitant AF, approximately 80% of whom had pre-existing AF [88]. Patients with alternative indications for therapeutic anticoagulation were excluded, and more than one-third received therapeutic parenteral anticoagulation. In propensity score-matched analysis, therapeutic parenteral anticoagulation was not associated with fewer ischemic strokes (1.3% vs 1.4%; RR 0.94, 95% CI 0.77–1.15) but was associated with more clinically significant bleeding (8.6% vs 7.2%; RR 1.21, 95% CI 1.10–1.32) [88]. Among patients with pre-existing AF, anticoagulation was likewise not associated with reduced ischemic stroke (RR 1.12, 95% CI 0.86–1.44), while bleeding risk was increased (RR 1.23, 95% CI 1.10–1.36). However, these findings should be interpreted cautiously, as the primary exposure was therapeutic parenteral anticoagulation during sepsis rather than a predefined strategy of continuation versus interruption of long-term oral anticoagulation. Furthermore, no high-level evidence has evaluated this strategy in other ICU populations. Only small retrospective studies have similarly suggested an increased risk of bleeding when therapeutic anticoagulation is maintained, without evidence of protection against ATEs [40,[111], [112], [113]]. Further studies are needed to clarify the benefit-risk balance of maintaining therapeutic anticoagulation in critically ill patients with pre-existing AF.

## Author contributions

VL, SH, KG, and L-M J defined the questions. The answers to the questions were drafted jointly. Each person wrote the argument for the question(s) for which he or she was responsible and had it validated by the group. VL harmonized the writing of the different parts. All authors reviewed, corrected, and approved the final text.

## Consent for publication

Not applicable.

## Funding

The organization of these guidelines was funded by the French Intensive Care Society.

## Ethics approval and consent to participate

Not applicable.

## Competing interests

VL: grants from Leopharma and personal fees from AOP Health; NA: no competing interest; PA: no competing interest; FB: no competing interest; SB: no competing interest; SE: no competing interest; JH: no competing interest; SH: no competing interest; JJ: lecture fees from AOP Health; AJ: no competing interest; KK: no competing interest; AMD reported grants and personal fees from LEO Pharma; SP reports personal fees from AOP-health and Viatris for lectures; LMJ: no competing interest.

## Availability of data and materials

Not applicable
