# Supplementary material for: Guidelines for the management of atrial fibrillation in adult patients admitted to an intensive care unit
Source: Ann Intensive Care. 2026 Sep 14;16:100159. doi: 10.1016/j.aicoj.2026.100159 (PMC13676918; doi:10.1016/j.aicoj.2026.100159)
Supplement: Supplementary file 1 [file mmc1.docx]

Appendix 1: PICO Questions

Question 1

In intensive care unit (ICU) patients with new-onset atrial fibrillation (NOAF), should transthoracic echocardiography be systematically performed?

Question 2

In ICU patients with NOAF, should transesophageal echocardiography be systematically performed?

Question 3

In ICU patients with NOAF, should fluid boluses be systematically performed?

Question 4

In ICU patients with NOAF, should magnesium be systematically administered?

Question 5

In ICU patients with NOAF requiring cardioversion, should clinicians target a serum potassium level of at least 4.5 mmol/L or a level within the laboratory reference range?

Question 6

In ICU patients with NOAF, should TSH testing be performed systematically?

Question 7

In ICU patients with NOAF during cardiogenic shock, should physicians favor a rhythm-control strategy or a rate-control strategy, in addition to the management of potentially reversible triggers?

Question 8

- 8A: In ICU patients with NOAF following cardiac surgery without hypotension or acute pulmonary edema, should physicians favor a rhythm-control or a rate-control strategy, in addition to the management of potentially reversible triggers?
- 8B: Outside the postoperative cardiac surgery setting, in ICU patients with NOAF without hypotension or acute pulmonary edema, should physicians favor rhythm-control or rate-control strategy, in addition to the management of potentially reversible triggers?

Question 9

In ICU patients with NOAF during septic shock, should a rhythm-control or a rate-control be initiated in addition to the management of potentially reversible triggers, compared with the management of potentially reversible triggers alone?

Question 10

- 10A: In ICU patients with NOAF and concomitant cardiogenic shock or acute pulmonary edema requiring invasive mechanical ventilation who require urgent restoration of sinus rhythm, should physicians favor electrical or pharmacological cardioversion?
- 10B: In ICU patients with NOAF and concomitant acute pulmonary edema not requiring invasive mechanical ventilation who require urgent restoration of sinus rhythm, should physicians favor electrical or pharmacological cardioversion?

Question 11

- 11A: In ICU patients with NOAF and sepsis without severe left ventricular systolic dysfunction, should amiodarone be preferred over other antiarrhythmic drugs as first-line pharmacological agent to restore sinus rhythm?
- 11B: In other ICU patients with NOAF, should amiodarone be preferred over other antiarrhythmic drugs as first-line pharmacological agent to restore sinus rhythm?

Question 12

- 12A: In ICU patients with NOAF with cardiogenic shock or acute pulmonary edema for whom pharmacological cardioversion with amiodarone has been decided upon, should the intravenous route be preferred over the oral route?
- 12B: In ICU patients with NOAF without cardiogenic shock or acute pulmonary edema for whom pharmacological cardioversion with amiodarone has been decided upon, should the intravenous route be preferred over the oral route?

Question 13

When a rate control has been decided upon in patients with pre-existing AF or NOAF who have neither cardiogenic shock requiring vasopressor and/or positive inotropic agent nor left and/or right ventricular systolic dysfunction, should Beta-blockers be preferred over other classes of rate-control drugs?

Question 14

When rate control has been decided upon in patients with pre-existing AF or NOAF who have cardiogenic shock requiring vasopressor and/or positive inotropic agents, or left and/or right ventricular systolic dysfunction, should Amiodarone be preferred over other classes of rate-control drugs?

Question 15

- 15A: In ICU patients with NOAF lasting less than 48 h who are not undergoing cardioversion, should therapeutic anticoagulation be initiated?
- 15B: In ICU patients with NOAF lasting at least 48 h who are not undergoing cardioversion, should therapeutic anticoagulation be routinely administered?

Question 16

In ICU patients with NOAF undergoing cardioversion, should therapeutic anticoagulation be administered during the peri-cardioversion period?

Question 17

In ICU patients with NOAF for whom therapeutic anticoagulation has been decided upon, should unfractionated heparin be preferred over low-molecular-weight heparin?

Question 18

In ICU patients with NOAF who have returned to sinus rhythm and in whom therapeutic anticoagulation has been initiated, should prolonged therapeutic anticoagulation (four weeks) be preferred over early discontinuation of therapeutic anticoagulation?

Question 19

In ICU patients with pre-existing AF, except for those at very high thrombotic risk (e.g., multiple previous strokes, a mechanical valvular prosthesis, mitral stenosis, or an intracardiac thrombus), should therapeutic anticoagulation be continued rather than discontinued?
